# Combined Effects of Lipophilic Phycotoxins (Okadaic Acid, Azapsiracid-1 and Yessotoxin) on Human Intestinal Cells Models

**DOI:** 10.3390/toxins8020050

**Published:** 2016-02-19

**Authors:** Pierre-Jean Ferron, Kevin Dumazeau, Jean-François Beaulieu, Ludovic Le Hégarat, Valérie Fessard

**Affiliations:** 1 Toxicology of Contaminants Unit, French Agency for Food, Environmental and Occupational Health & Safety, Fougères 35300, France; kevin.dumazeau@gmail.com (K.D.); ludovic.lehegarat@anses.fr (L.L.H.); valerie.fessard@anses.fr (V.F.); 2 Laboratory of Intestinal Physiopathology, University of Sherbrooke, Sherbrooke, QC J1G 0A2, Canada; jean-francois.beaulieu@usherbrooke.ca

**Keywords:** lipophilic phycotoxins, cytotoxicity, enterocytes, mixtures

## Abstract

Phycotoxins are monitored in seafood because they can cause food poisonings in humans. Phycotoxins do not only occur singly but also as mixtures in shellfish. The aim of this study was to evaluate the *in vitro* toxic interactions of binary combinations of three lipophilic phycotoxins commonly found in Europe (okadaic acid (OA), yessotoxin (YTX) and azaspiracid-1 (AZA-1)) using the neutral red uptake assay on two human intestinal cell models, Caco-2 and the human intestinal epithelial crypt-like cells (HIEC). Based on the cytotoxicity of individual toxins, we studied the interactions between toxins in binary mixtures using the combination index-isobologram equation, a method widely used in pharmacology to study drug interactions. This method quantitatively classifies interactions between toxins in mixtures as synergistic, additive or antagonistic. AZA-1/OA, and YTX/OA mixtures showed increasing antagonism with increasing toxin concentrations. In contrast, the AZA-1/YTX mixture showed increasing synergism with increasing concentrations, especially for mixtures with high YTX concentrations. These results highlight the hazard potency of AZA-1/YTX mixtures with regard to seafood intoxication.

## 1. Introduction

Phycotoxins are secondary metabolites produced by some phytoplanktonic unicellular algae. Due to their accumulation in filtering shellfish, several acute food intoxications following human consumption of contaminated shellfish have been well documented [1]. Phycotoxins are classified into hydrophilic and lipophilic groups according to their chemical properties. The EFSA (European Food and Safety Authority) report on marine biotoxins established five classes of toxins in the lipophilic group: okadaic acid (OA) and its analogs, azaspiracids (AZAs), pectenotoxins (PTXs), yessotoxins (YTXs) and spirolides (SPX) [2]. While the effects of OA and AZAs caused in humans have been well described, the effects caused by PTX-2, YTX and SPX still remain unclear, since there is no conclusive proof of human intoxication [1,3]. OA and its analogs, the dinophysistoxins, are polyethers responsible for the diarrhetic shellfish poisoning (DSP), causing symptoms such as nausea, severe diarrhea and abdominal pain [4]. These toxins are found worldwide and are protein phosphatase (PP) inhibitors (primarily PP2A, and to a lesser extent PP1 [5]. AZAs are composed of a spiro-ring assembly, a cyclic amino group and a carboxylic function. They are responsible for azaspiracid poisoning (AZP) [6] with symptoms similar to DSP (nausea and diarrhea), except that neurotoxicity has also been reported during *in vivo* experiments in mice [7]. Azaspiracids are relatively less common in seafood and have been described only sporadically in some areas in Europe [6], North America [8], Asia [9] and in northwestern Africa [10]. Nevertheless, the major AZA-producing species *Azadinium* sp. [11] has a widespread geographical distribution [11,12], and reports on new toxic species and new AZA analogs are increasing [13]. Although the mode of action of the AZA group has not been elucidated, the AZA toxins have been shown to inhibit endocytosis [14] and induce cytoskeleton disorganization [15]. Yessotoxins (YTXs) are sulfated polyether toxins with a hydrophobic tail and are found in shellfish all over the world [16]. The mode of action of YTXs has not yet been characterized, but YTX is known to induce endoplasmic reticulum stress [17], apoptosis [18], and endocytosis inhibition [19]. OA, AZA and YTX analogs can co-occur in shellfish [20,21,22]. Since no data are available on the toxicity of OA, AZA-1 and YTX mixtures in humans, EFSA has recommended to investigate the effects of these possible combinations [2]. Thus far, the effects of binary mixtures of OA and YTX [23], OA and AZA-1 (Azaspiracid-1) [24], and YTX and AZA-1 [25], have been studied in mice. However, none of these studies showed toxic interactions. As *in vivo* studies require laboratory animals and do not enable studying a large range of mixtures combinations, we therefore investigated the combined effects of lipophilic toxins using an *in vitro* approach in order to supplement previous results obtained *in vivo*. OA, AZA-1 and YTX have been shown separately to induce alterations in the mouse intestine [7,26,27]. Therefore, we conducted *in vitro* studies on human intestinal cells. Caco-2 cells were selected as a model for human enterocytes, according to the OA toxicity results already available on this cell line [28,29] as well as for AZA-1 and YTX toxicity [30,31]. To address the combined effects on a non-cancerous human intestinal cell line, the human intestinal epithelial crypt-like cells (HIEC), the non-transformed, non-immortalized cell model isolated from intestines of human embryos [32] was selected. This cell model, under specific conditions, expresses specific crypt-cell markers, and can be maintained in culture with differentiated characteristics for up to 30 passages [33].

## 2. Materials and Methods

### 2.1. Cell Cultures

The Caco-2 cells HTB-37 were obtained from ATCC (American Type Culture Collection, LGC Standards, Molsheim, France). Cells were cultured in MEM medium containing 10% fetal calf serum (FCS), 1% non-essential amino acids, 50 U/mL penicillin and 50 μg/mL streptomycin (Thermo Fisher Scientifc, Illkrich, France).

The HIEC cells were donated by Jean-François Beaulieu, previously described by [32]. HIEC were cultivated in OPTI-MEM medium (Thermofisher Scientific, Illkrich, France), supplemented with 20 mM HEPES (Thermofischer Scientific), 10 mM GlutaMAX (Thermofisher Scientific), 10 ng/mL EGF (Thermofisher Scientific) and 5% fetal bovine serum (Cellect Gold; MP Biomedicals, Aurora, OH, USA). HIEC cells exhibit all the morphological and functional characteristics of normal human proliferative crypt cells and are considered to be undifferentiated crypt-like progenitor cells [34,35].

For assays, HIEC and Caco-2 cells were seeded at a density of 30,000 cells/cm^2^ and 90,000 cells/cm^2^, respectively, in Nunc 96-well microplates (Thermo Fisher Scientific, Illkrich, France), 24 h prior to exposure.

### 2.2. Toxins

OA, AZA-1 and YTX were purchased from IMB/NRC (Halifax, NS, Canada). For assays, toxins were prepared in culture medium without FCS.

### 2.3. Neutral Red Uptake Assay

Neutral red powder (N4638) was purchased from Sigma-Aldrich (Lyon, France) and 0.1% neutral red solution was prepared in Caco-2 and HIEC culture media. Following treatment with toxins, cells were rinsed with PBS, and 100 µL of neutral red solution was added to each well and incubated for 2 h at 37 °C. Cells were then rinsed with PBS and 100 µL of solubilization solution (1% acetic acid in 50% ethanol) was added to each well. Absorbance was read at 540 nm and viability was calculated as percentage of mean absorbance (at least three independent experiments) relative to the control cells (growth medium with 5% MeOH).

### 2.4. Combinations of Toxins

Cytotoxicity of combined phycotoxins was studied for three binary mixtures: OA and YTX, OA and AZA-1 and AZA-1 and YTX (Table 1). For each combination of toxins, four different ratios of toxins were tested, ranging from a ratio with high potency of one toxin to a ratio with high potency for the other. The chosen toxin ratios were based on preliminary individual cytotoxicity experiments.

### 2.5. Data Analysis

*Cytotoxicity of individual phycotoxins.* The dose-response cytotoxicity of each phycotoxin was modeled using a Hill slope model on GraphPad Prism 5 (GraphPad Software, La Jolla, USA, 2010). If the coefficient *m*, indicative of the shape of the dose-response curve, was >1 (sigmoid curve) and if the linear correlation coefficient of the model was greater than or equal to 0.9, the inhibitory concentration 50% (IC_50_) was calculated.

*Cytotoxicity of phycotoxin mixtures*. For each phycotoxin, the dose–response cytotoxicity was modeled using the median-effect equation of the mass-action law described by Chou and Talalay [36]. The median-effect equation is defined by:

(1) log(fafu)=mlogD−mlogDm

where *D* is the dose, *D_m_* is the dose required for 50% inhibition, *Fa* is the fraction affected by the dose *D*, *m* is a coefficient of the sigmoidicity of the dose effect, and *Fu* = 1 – *Fa.*

The combination index (*CI*) method was used to analyze interactions between phycotoxins. Following Chou and Talalay (1984) [36], the *CI* method was calculated as follows:

$$CI=\frac{{\left(D\right)}_{1}}{{\left(Dm\right)}_{1}}+\frac{{\left(D\right)}_{2}}{{\left(Dm\right)}_{2}}$$

where (*D*)_1_ and (*D*)_2_ are the doses of the two mixed phycotoxins, and (*Dm*)_1_ and (*Dm*)_2_ are the doses of the individual phycotoxins, respectively. *CI* < 1, *CI* = 1 and *CI* > 1 indicate if the mixture displays a synergistic, additive or antagonistic effects, respectively. *CI* values were established for cytotoxicity percentages ranging from 5% to 95% (corresponding to *Fa* values ranging from 0.05 to 0.95). For *Fa* values of 0.25, 0.50 and 0.75, we determined the dose reduction index (DRI) values. The DRI is a measure of how many fold the dose of each drug in a synergistic combination is reduced, for a given effect level, compared with the doses of each drug alone. The DRIs were only calculated when the *CI* values indicated synergism. The dose-response analyses of phycotoxin mixtures and the *CI*/DRI calculation were performed using CompuSyn software, version 3.01 (ComboSyn Inc., Paramus, NJ, USA).

## 3. Results

### 3.1. Individual Cytotoxicity of OA, AZA-1 and YTX

HIEC and Caco-2 cells were exposed to phycotoxins OA, AZA-1 and YTX for 24 h. The three phycotoxins showed a dose-dependent toxic effect on both cell lines (Table 1). On Caco-2 cells, toxins displayed IC_50_ values of 78.5 nM for OA, 4.0 nM for AZA-1 and 4.1 nM for YTX. On HIEC, toxins displayed IC_50_ values of 65.3 nM for OA, 12.5 nM for AZA-1 and 3.4 nM for YTX. The results for OA and YTX toxicity on the two cell types were similar, but AZA-1 IC_50_ values were significantly different in HIEC and Caco-2 cells, with a greater effect observed on Caco-2 cells.

### 3.2. Combined Cytotoxicity of AZA-1 and YTX

**Figure 1:**
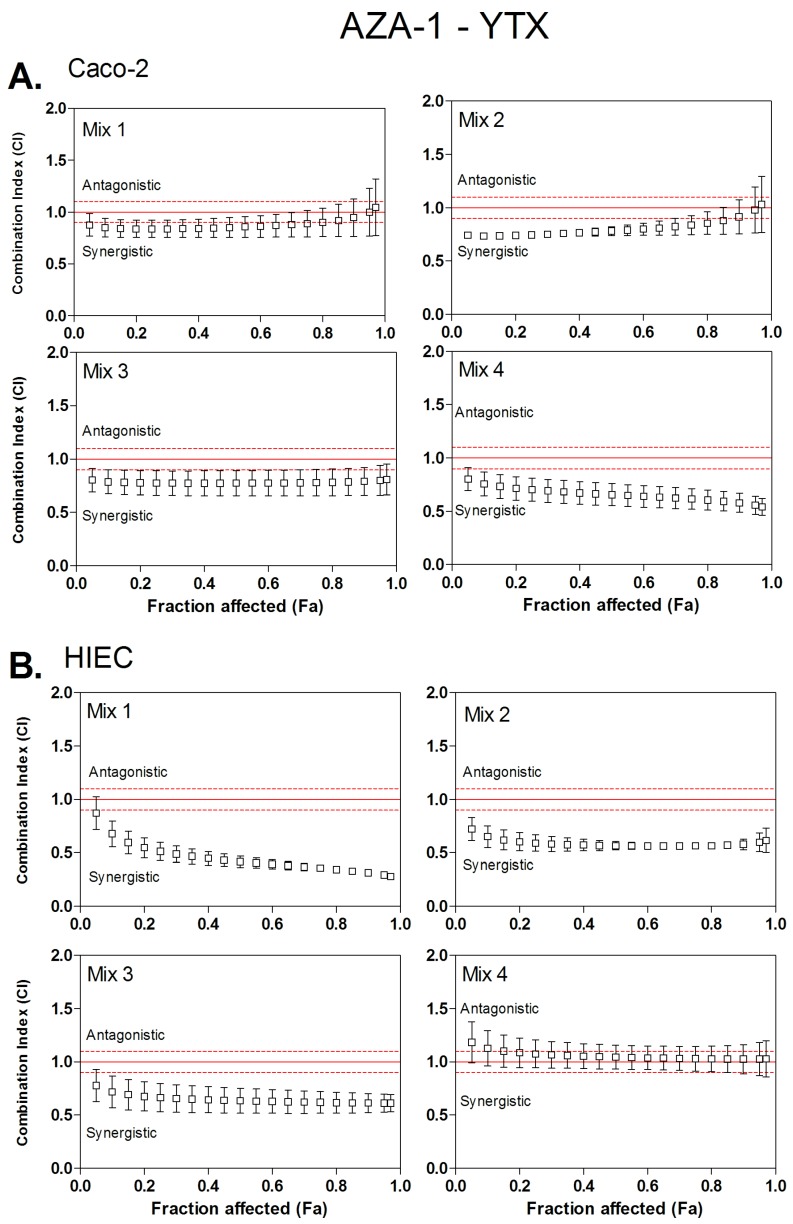
Combination index (*CI*) plot for binary mixtures of azaspiracid-1 (AZA-1) and yessotoxin (YTX) in four different molar ratios. *CI* values were calculated from fractional cytotoxicity (*Fa*) by computer modeling (CompuSyn) on *Fa* = 0.05 to 0.95. *CI* < 1, *CI* = 1 and *CI* > 1 indicate synergistic, additive and antagonistic effects, respectively. The vertical bar indicates the standard deviation for the three replicates. Dashed lines indicate upper and lower limit of additivity [36]. (**A**) Combinary index plots based on Caco-2 cells experiments; (**B**) Combinary index plots based on HIEC cells experiments.

**Figure 2:**
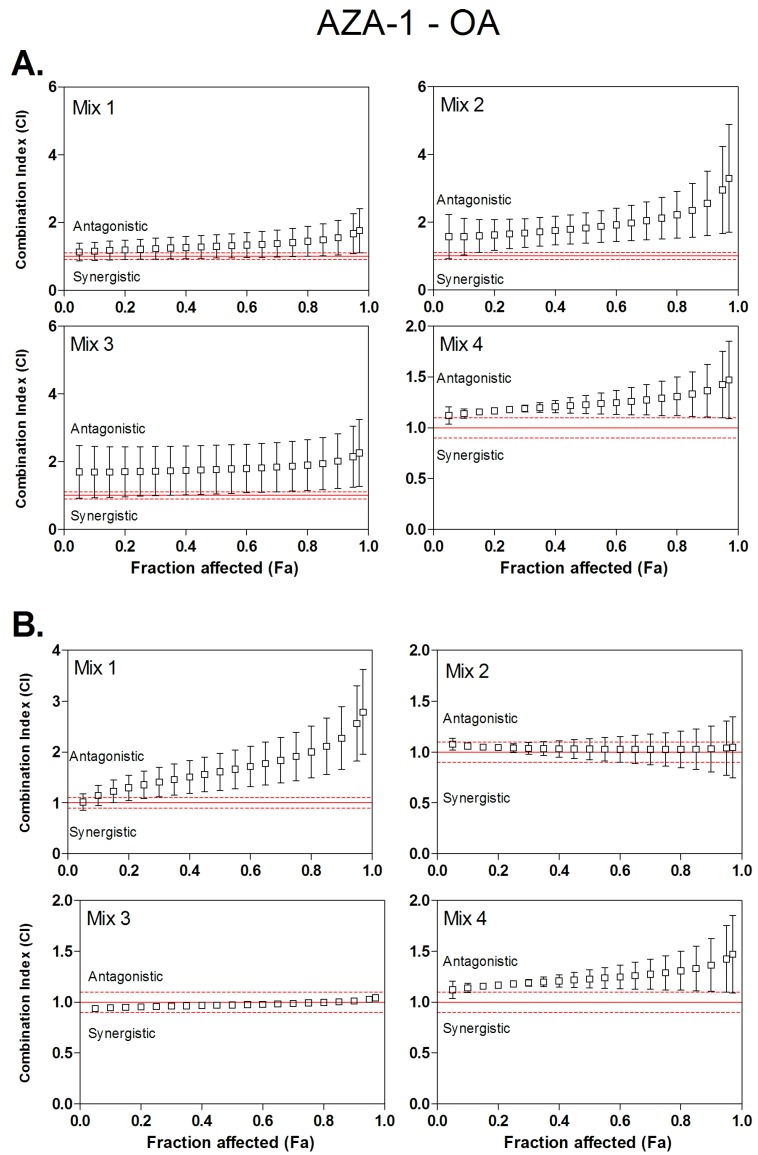
Combination index plot for binary mixtures of okadaic acid (OA) and azaspiracid-1 (AZA-1) at four different molar ratios. *CI* values were calculated from fractional cytotoxicity (*Fa*) by computer modeling (CompuSyn) on *Fa* = 0.05 to 0.95. *CI* < 1, *CI* = 1 and *CI* > 1 indicate synergistic, additive and antagonistic effects, respectively. The vertical bar indicates the standard deviation for the three replicates. Dashed lines indicate upper and lower limit of additivity [36]. (**A**) Combinary index plots based on Caco-2 cells experiments; (**B**) Combinary index plots based on HIEC cells experiments.

**Figure 3:**
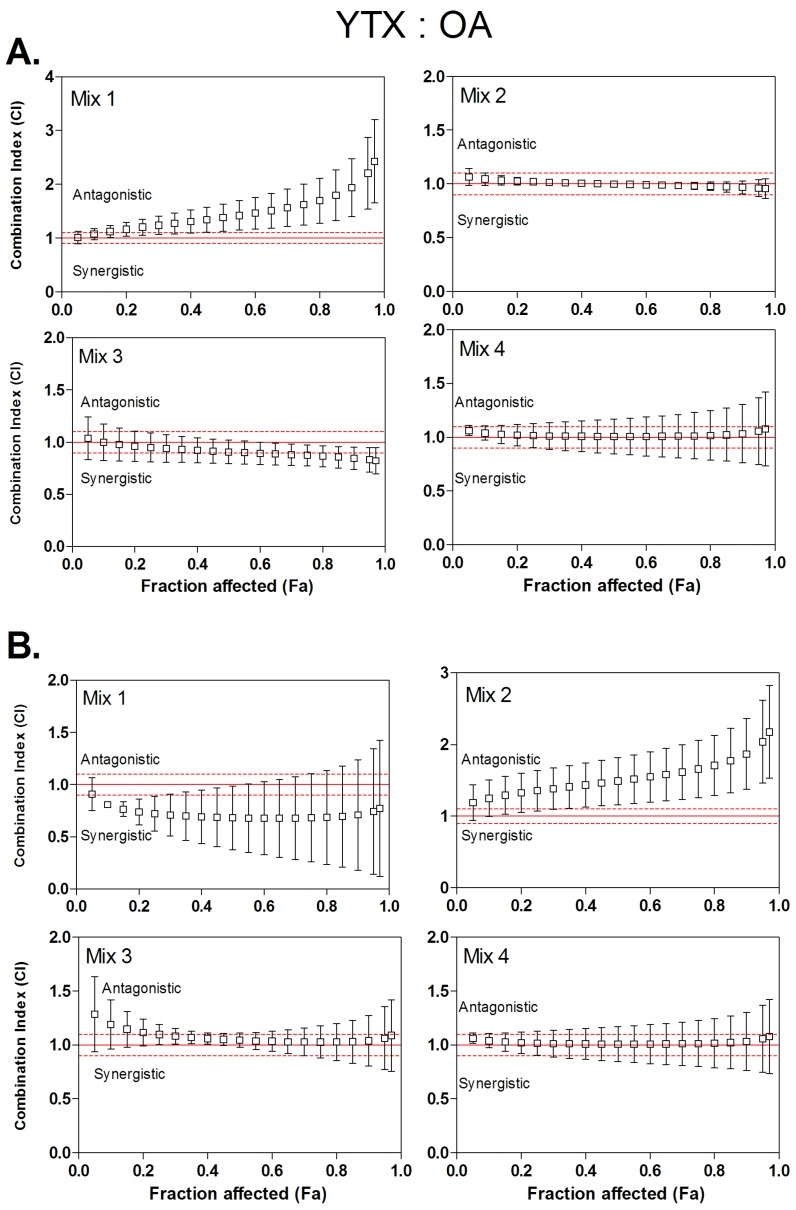
Combination index (*CI*) plot for binary mixtures of okadaic acid (OA) and yessotoxin (YTX) at four different molar ratios. *CI* values were calculated from fractional cytotoxicity (*Fa*) by computer modeling (CompuSyn) on *Fa* = 0.05 to 0.95. *CI* < 1, *CI* = 1 and *CI* > 1 indicate synergistic, additive and antagonistic effects, respectively. The vertical bar indicates standards deviation between the three experiments. Dashed lines indicate upper and lower limit of additivity [36]. (**A**) Combinary index plots based on Caco-2 cells experiments; (**B**) Combinary index plots based on HIEC cells experiments.

The type of interaction (synergism, additivity or antagonism) for binary mixtures of OA, AZA-1 and YTX were characterized using the *CI* method. Four ratios of toxins were tested using the neutral red uptake assay (Table 2). These ratios were arbitrary defined, according to IC_50_ values of individual toxins. As all toxins are not equally potent, molecular ratios were determined in order that, in a binary combination of two toxins A and B, two mixtures will enhance toxicity mainly due to toxin A, and the two others will enhance toxicity mostly due to toxin B. Then, *CI* values were calculated from *Fa* values of 0.05 (corresponding to IC_05_) to 0.95 (corresponding to IC_95_) (Figure 1, Figure 2 and Figure 3). Dose-reduction indices (DRI) were calculated when synergism (*CI* < 1) was observed (Table 3).

AZA-1/YTX mixtures displayed a dose-dependent cytotoxicity on both Caco-2 and HIEC cells. Toxins interactions were analyzed using the CI method applied on cytotoxicity data (Figure 1). Figure 1A suggests that, in Caco-2 cells, in mixtures in which toxicity is mainly due to the presence of AZA-1, mixture 1 displayed additivity, while mixture 2 showed only a slight synergistic effect between IC_10_ and IC_75_ with a CI value ranging from 0.74 to 0.88. For mixtures 3 and 4 with a toxic effect mainly induced by the presence of YTX, mixture 3 showed a slight synergistic effect, and even more pronounced in mixture 4. Figure 1B suggests that a synergistic effect occurred also in HIEC, and was mainly caused by mixtures where AZA-1 is the main toxic compound (Mix 1 and 2), and to a lesser degree with mixture 3 where YTX is the main toxic compound. The synergistic effect was even more pronounced as CI level reached a value of 0.3. In general, CI values ranged between 0.9 and 0.3, and synergism was greater at high toxic potency (*Fa* > 0.5). Conversely to Caco-2 cells, mixture 4 displayed additivity in HIEC cells.

The DRI values in Table 3 indicate for each toxin the fold reduction in the IC_25_, IC_50_ and IC_75_ values to apply in order to obtain the theoretical additive effect. Except for mixture 4 in HIEC cells and mixture 1 in Caco-2 cells, all mixtures displayed a synergistic effect. The combination index method allows calculating a theoretical dose reduction index, which can be applied as a factor on toxins concentration when an additive effect is considered. The DRI values for YTX in both Caco-2 and HIEC is higher to the DRI values for AZA-1, suggesting that, in the mixture, YTX is more potent than AZA-1.

### 3.3. Combined Cytotoxicity of AZA-1 and OA

AZA-1/OA mixtures displayed dose-dependent cytotoxicity on Caco-2 and HIEC cells. Toxins interactions were analyzed using the CI method applied on cytotoxicity data (Figure 2). Figure 2 suggests that mixtures of AZA-1 and OA displayed principally antagonistic cytotoxicity in Caco-2. Indeed, in Figure 2A, all mixtures, 1, 2, 3 and 4, show CI values ranging between 1.2 and 3.3 in Caco-2. In HIEC cells (Figure 2B), when the mixture is driven by one of the two toxins (mixture 1, where toxicity is mainly due to the presence of AZA-1, and mixture 4 where the toxicity is mainly due to the presence of OA), an antagonistic cytotoxicity was observed with *CI* values between 1 and 2.8. However, for mixtures 2 and 3, the toxic effects of the two toxins were additive (*CI* between 0.8 and 1.2).

### 3.4. Combined Cytotoxicity of YTX and OA

YTX/OA mixtures displayed dose-dependent cytotoxicity on both Caco-2 and HIEC cells. Figure 3 suggests that mixtures of YTX and OA displayed different interaction profile depending on cell types and mixture composition. In Caco-2 cells (Figure 3A), mixtures 2–4 displayed additive effects, showing *CI* values ranging between 0.8 and 1.2. In mixture 1, where the toxic potency is driven by YTX, the combined effect was strongly antagonistic (*CI* values up to 2) at high toxin concentrations (*Fa* above 0.8).

In HIEC cells (Figure 3B), for mixtures 3 and 4, where the toxic potency is driven by OA, the combined cytotoxic effects were additive, with *CI* values ranging between 1 and 1.28. However, for mixture 1, where the toxic potency is driven by YTX, the combined effect was synergistic, (*CI* values between 0.9 and 0.67) but with a large variability. On the contrary, for mixture 2, even if the toxicity is mainly due to high YTX concentration, an antagonistic effect was detected with *CI* values increasing from 1.2 at Fa 0.05 to 2.1 at *Fa* 0.95.

## 4. Discussion

Phycotoxins are known to accumulate in shellfish, which may cause human seafood poisoning [2]. In 2008, Wang estimated that algal toxins caused more than 50,000–500,000 intoxication incidents per year, with an overall mortality rate of 1.5% [37]. Data indicate that lipophilic phycotoxins, including the OA, AZA and YTX groups, can co-occur in shellfish [20,21]. To date, risk assessment has been based on toxicity data from individual toxins; however, to address the issue of co-occurrence, the EFSA recommends studying the toxic effects of lipophilic phycotoxins in mixtures [2]. We therefore studied the interactions of binary mixtures of OA, AZA-1 and YTX on human intestinal cell models. Cytotoxicity of OA, AZA-1 and YTX mixtures was investigated using the neutral red uptake assay on the conventional intestinal model Caco-2 cell line and the intestinal cell line HIEC, which has only recently been used in toxicology studies [38].

OA, AZA-1 and YTX are cytotoxic towards Caco-2 and HIEC cells. The IC_50_ of OA on HIEC and Caco-2 cells (65.29 nM and 78.52 nM, respectively) was close to the one we previously established on Caco-2 (49,67 nM [29]. The neutral red assay is based on the incorporation and binding of the supravital dye neutral red in the lysosomes. Therefore, the response of Caco-2 cells towards AZA-1 exposure using the neutral red assay, compared to the lack of response observed when other cytotoxicity assays are performed on this cell line [8,39], indicates that lysosomes may play a key role in AZA-1 cytotoxicity. Our results also showed that YTX is highly toxic on both intestinal cell lines, in contrast with the low toxicity observed in rodents *in vivo*. Previously, YTX has been reported to induce no toxic effects on the murine intestine neither by 10 mg/kg oral dose nor by lethal intraperitoneal doses [24]. Furthermore, the consumption of shellfish contaminated with YTX has never been involved in any case of human poisoning. Among the three toxins, YTX was the most potent toxin on HIEC cells, whereas YTX and AZA-1 showed equal toxicity on Caco-2 cells. Our *in vitro* results on intestinal cell models do not match with the toxic potency observed upon oral administration to mice. While OA and AZA-1 displayed a Lethal Dose 50 (LD_50_) of 0.5–2 mg/kg on mice, with an obvious toxicity on the intestine [27,40,41], YTX did not cause any death on mice, or intestinal toxicity at ≤10 mg/kg [42]. The toxicity of YTX detected on proliferative intestinal cells is probably driven by a specific pathway. It has been established that the kinase pathways, especially the MEK/ERK pathway, play an essential role in human intestinal cell survival [43]. JNK, and the kinase pathway induced by YTX in parapoptosis cell death may be involved in YTX cytotoxicity observed in human intestinal proliferative cells [44].

In our study, the combined effects of the binary mixtures of OA, AZA-1 and YTX were assessed with the *CI* method [36] using four different toxin ratios. The ratios of phycotoxins in shellfish is unpredictable [21]; we therefore tested several ratios for each toxin mixture to cover a wide range of contamination possibilities. The *CI* values were calculated for different toxicity levels to quantify synergistic or antagonistic toxin interactions. When synergism was detected, a DRI value was determined for each toxin in the mixture [45]. Most of the combined effects of binary mixtures of phycotoxins were additive or antagonistic on Caco-2 and HIEC cells at low (IC_25_) or high (IC_75_) toxin doses. A synergistic effect was only observed with AZA-1 and YTX combinations in both cell lines.

Our results showed that the mixtures of YTX and OA induced rather additive and in some cases antagonistic effects. These data confirm the observation reported from oral exposure of mice where no combined toxic effect was detected after repeated oral doses of 1 mg/kg YTX and 0.185 mg/kg of OA in comparison with individual toxins [23]. Similarly, Rodriguez *et al.* (2015) [46] studied recently the toxicity of OA (1–500 nM) in combination with 500 nM YTX on human neuroblastoma cells but did not observed any evidence of synergism after 24 h of exposure although an increased toxicity was detected after 48 h of exposure. Therefore, we suggest that further investigations with a longer incubation time should be performed using our method as a longer treatment time could modify interactions and might induce different pathways.

While OA/AZA1 showed in our study rather an additive effect for the mixtures 2 and 3, an antagonistic effect was observed in HIEC cells when the mixtures contain a high level of one of the toxin (mixtures 1 and 4). Similarly, mice orally exposed to mixtures with up to 570 µg/kg AZA-1 and 880 µg/kg OA did not display any combined effects compared with mice exposed to individual toxins. Furthermore, when OA and AZA-1 are given simultaneously, their uptake through the gastrointestinal (GI) epithelium, as well as their toxicity in the GI tract, decreased [24,40]. Both our results and the ones obtained *in vivo* suggest that the presence of both OA and AZA-1 in contaminated shellfish may result in additive toxicity for consumers.

AZA-1/YTX mixtures displayed a synergistic effect for most of the combinations tested. The DRI was calculated to determine the dose-reduction for AZA-1 and YTX in a synergistic combination at IC_25_, IC_50_ and IC_75_ [47]. For AZA-1/YTX mixtures, the DRI ranged from 1.7 to 10.9 and 1.3 to 10.4 in HIEC and Caco-2 cells, respectively, indicating a similar degree of synergy in both cell lines. For the risk assessment of mixtures, the calculation of magnitude of interaction is an important parameter [48]. Studies performed on mice have shown that oral administration of YTX (1 or 5 mg/kg) combined with 200 µg/kg AZA-1 did not enhance the oral toxicity of AZA-1 alone. Similarly, absorption of YTX or AZA-1 was not increased when both toxins were administered together [25]. However, our results indicate that co-exposure of human intestinal cells to AZA-1 and YTX may induce higher toxicity than exposure to just one toxin. Indeed, AZA-1 and YTX share a common toxicological mechanism, namely cellular endocytosis. AZA-1 and YTX induce the accumulation of ECRA100 in MCF-7 cells at the same level of potency (1 mM) [31]; however, AZA-1 inhibits the formation of the late endosomes, whereas YTX alters an ulterior step of protein degradation [14]. Co-exposure to AZA-1 and YTX may thus strongly inhibit cellular endocytosis. This complementary effect on intracellular protein trafficking may explain the toxicological synergism of AZA-1 and YTX on HIEC and Caco-2 cells.

The present study showed that Caco-2 and HIEC cells are sensitive to OA, AZA-1 and YTX, with an acute toxicity dose-response. Although the IC_50_ values of OA and YTX were quite similar on the two cell lines, AZA-1 displayed higher toxic potency on HIEC cells. In contrast to previous *in vivo* results, YTX was a very active compound on both human intestinal cell lines, suggesting that a specific pathway could be involved in *in vitro* human models. The CI method was used to predict the effect of binary mixtures of phycotoxins. As observed *in vivo*, the effects of OA/AZA-1 and OA/YTX mixtures on human intestinal cells are rather additive and eventually antagonistic in few cases. However, the AZA-1/YTX mixture showed a synergistic interaction suggesting an additional risk when consuming co-contaminated shellfish with these toxins. However, our *in vitro* conclusion cannot be easily extrapolated to *in vivo* situations. As the CI-isobologram method requests a large number of assays to allow good computer modeling, further studies should be conducted *in vivo* to study AZA-1 and YTX interactions, using such mathematical model. The CI-isobologram method can quantify the interactions of the toxicological effects in mixtures of up to five drugs [45]. Because phycotoxins frequently occur in mixtures in shellfish meat [21,22], evaluation of toxicological interactions between lipophilic phycotoxins OA, AZA, PTX-2 and YTX in tertiary and quaternary combinations is the next step in phycotoxin mixture studies. Similar experiments have been conducted on mycotoxin mixtures and have shown that low doses of mycotoxins in food and diet may be more toxic than predicted from individual mycotoxins [49].

## 5. Conclusions

Based on neutral red uptake assay, OA, AZA-1 and YTX induced cytotoxicity on both Caco-2 and HIEC cell lines. Binary combinations of OA and AZA-1 and OA and YTX showed an additive and, in few cases, an antagonistic effect using the concept of combination index on our models. However, data analysis claimed synergism between AZA-1 and YTX on both Caco-2 and HIEC cells. Our *in vitro* findings could not enable any extrapolation to *in vivo* situations without further investigation, including a clear characterization of toxins mechanisms of action in mixtures.

## Figures and Tables

**Table 1:** Cytotoxicity of okadaic acid (OA), azaspiracid-1 (AZA-1) and yessotoxin (YTX) determined using the neutral red uptake assay on Caco-2 and HIEC cells after 24 h exposure. Inhibitory concentration 50% (IC_50_) values of OA, AZA-1 and YTX in Caco-2 and HIEC cells from the neutral red uptake assay. IC_50_ values and 95% Confidence Interval (CI) were calculated from six independent experiments performed in triplicate.

Cytotoxicity	Caco-2			HIEC		
	IC50 (nM)	95% CI	*n*	IC50 (nM)	95% CI	*n*
OA	78.52	46.48–111.60	6	65.29	49.14–81.45	6
AZA-1	4.03 *	2.05–6.01	6	12.52 *	8.64–16.40	6
YTX	4.08	1.18–7.83	6	3.36	1.17–5.93	6

Symbol * indicate a significant difference at *p* < 0.05 between the IC_50_ values of the two cell lines using a *t* test.

**Table 2:** Molar ratios of toxin concentrations used in binary mixtures.

Toxins Mixture	Molar Combination Ratio			
	Mix 1	Mix 2	Mix 3	Mix 4
AZA:YTX	1:0.8	1:1.3	1:2.4	1:3.6
AZA:OA	1:51	1:27.2	1:15.3	1:8.2
YTX:OA	1:26.5	1:14.1	1:7.9	1:4.2

**Table 3:** Combination index (*CI*) values and dose-response index (DRI) values for binary mixtures of okadaic acid (OA), azaspiracid-1 (AZA-1) and yessotoxin (YTX) in Caco-2 and HIEC cells. CI values were calculated as described in Materials and Methods. DRI values were only calculated for toxin mixtures when synergistic effects were detected. DRI values were calculated for binary toxin mixtures by comparing the concentration required to reach IC_25_, IC_50_ and IC_75_ cytotoxicity. Values of DRI > 2 indicate synergistic effects.

Phycotoxins	Ratio	Caco-2						HIEC					
		IC25		IC50		IC75		IC25		IC50		IC75	
		CI	DRI	CI	DRI	CI	DRI	CI	DRI	CI	DRI	CI	DRI
AZA:YTX													
Mix 1	1:0.8	0.84	/	0.85	/	0.88	/	0.51	3.1:2.8	0.42	3.4:4.3	0.36	3.8:6.8
Mix 2	1:1.3	0.74	3.9:2.1	0.78	2.0:4.0	0.83	/	0.59	2.3:3.9	0.57	2.3:5.2	0.56	2.3:7.2
Mix 3	1:2.4	0.77	1.5:5.0	0.77	1.4:4.9	0.78	1.3:4.8	0.66	1.7:5.2	0.63	1.7:7.5	0.62	1.8:10.9
Mix 4	1:3.6	0.7	1.4:8.6	0.66	1.5:9.4	0.61	1.4:10.4	1.07	/	1.04	/	1.03	/
AZA:OA													
Mix 1	1:8.2	1.20	/	1.29	/	1.40	/	1.35	/	1.60	/	1.91	/
Mix 2	1:15.3	1.64	/	1.82	/	2.11	/	1.04	/	1.00	/	1.02	/
Mix 3	1:27.2	1.70	/	1.76	/	1.86	/	0.95	/	0.97	/	1.00	/
Mix 4	1:51	1.66	/	1.85	/	2.13	/	1.17	/	1.22	/	1.28	/
YTX:OA													
Mix 1	1:4.2	1.2	/	1.38	/	1.63	/	0.72	3.4:1.8	0.68	3.5:1.4	0.68	3.4:1.2
Mix 2	1:7.9	1.02	/	1.00	/	0.98	/	1.35	/	1.49	/	1.66	/
Mix 3	1:14.1	0.95	/	0.91	2.1:2.0	0.87	/	1.10	/	1.04	/	1.02	/
Mix 4	1:26.5	1.14	/	1.14	/	1.16	/	1.01	/	1.01	/	1.01	/
